# Chirurgische Therapie von Leber- und Pankreasmetastasen von Nierenzellkarzinomen

**DOI:** 10.1007/s00104-020-01331-3

**Published:** 2021-01-04

**Authors:** Astrid Bauschke, Annelore Altendorf-Hofmann, Aladdin Ali Deeb, Herman Kissler, Hans-Michael Tautenhahn, Utz Settmacher

**Affiliations:** Klinik für Allgemein‑, Viszeral- und Gefäßchirurgie, Universitätsklinikum Jena, Erlanger Allee 101, 07740 Jena, Deutschland

**Keywords:** Hepatopankreatische Metastasen, Chirurgie, Morbidität, Mortalität, Risikofaktoren, Hepato-pancreatic metastases, Surgery, Morbidity, Mortality, Risk factors

## Abstract

**Hintergrund:**

Der Stellenwert der chirurgischen Therapie hepatopankreatischer Metastasen des oligometastasierten Nierenzellkarzinoms ist Gegenstand der aktuellen Diskussion.

**Material und Methoden:**

Wir berichten über 51 Patienten, von denen 33 wegen Lebermetastasen und 19 wegen Pankreasmetastasen im Zeitraum von 1995 bis 2018 operiert wurden.

**Ergebnisse:**

Die 5‑Jahres-Überlebensrate aller Patienten nach Leberteilresektion war statistisch signifikant geringer (38 %, mediane Überlebenszeit 34 Monate) als nach Pankreasresektion (69 %, mediane Überlebenszeit 69 Monaten; *p* = 0,017). 21 Patienten haben bislang die Metastasenentfernung länger als 5 Jahr überlebt, 4 Patienten länger als 10 Jahre. Bei den R0-resezierten Patienten wurden Rezidive in 13 Fällen nach Leber- und 9 Fällen nach Pankreasresektion beobachtet. Die kumulative Rezidivrate nach 5 Jahren betrug bei der Leber 38 % und beim Pankreas 57 %. Bei R0-Leberteilresektionen erwiesen sich ein Intervall von <24 Monaten zwischen Nephrektomie und Leberresektion sowie multiple Metastasen als negative Prognosefaktoren.

**Diskussion:**

Unsere Ergebnisse gestatten eine aktive chirurgische Strategie in der Behandlung hepatopankreatischer Metastasen oligometastasierter Nierenzellkarzinome, insbesondere bei kompletter Resektion solitärer, metachroner Metastasen. Wiederholte Eingriffe bei komplett resektablen Metastasen führen zu langen tumorfreien Intervallen und tragen damit zu guten Langzeitergebnissen bei.

## Abstract (en)

**Background:**

The role of surgical treatment of hepato-pancreatic metastases from renal cell carcinoma is still under discussion.

**Material and methods:**

We report about 52 patients of whom 33 underwent surgery for liver metastases and 19 for pancreatic metastases from 1995 to 2018.

**Results:**

The 5‑year survival rate of all patients with partial liver resection was statistically significantly lower (38%, median survival time 34 months) than with pancreas resection (69%, median survival time 69 months, *p* = 0.017). Of the patients 21 survived the resection of metastases longer than 5 years and 4 patients longer than 10 years. In R0 resected patients, recurrences were observed in 13 cases after liver resection and in 9 cases after pancreas resection. The cumulative recurrence rate after 5 years was 38% for the liver and 57% for the pancreas. In R0 partial liver resections, an interval <24 months between nephrectomy and liver resection as well as multiple metastases were negative prognostic factors.

**Conclusion:**

In spite of high recurrence rates, surgical treatment for hepato-pancreatic metastases from renal cell carcinoma yielded very good long-term results, in particular with complete resection of solitary metachronous metastases. Repeated surgery for completely resectable metastases, resulted in long tumor-free intervals and thus contributed to good long-term results.

## Hintergrund

Metastasen des Nierenzellkarzinoms treten bei 20–30 % synchron und bei 50 % der Patienten metachron [4, 26] meist in Lunge, Knochen und Leber, aber selten im Pankreas auf [9, 14, 17]. So fanden sich in Autopsiestudien bei 40 % der Patienten Lebermetastasen sowie bei bis zu 12 % der Patienten Pankreasmetastasen [2]. Der Stellenwert der Resektion ggf. in Kombination mit lokal ablativen Verfahren in der Behandlung dieser Metastasen, insbesondere in der oligometastasierten Situation, im Vergleich zu anderen Verfahren (Radio‑, Hormon- oder medikamentöse Therapie mit einem Tyrosinkinaseinhibitor) ist bis heute Gegenstand von Diskussionen [25]. Die Europäische Gesellschaft für Urologische Leitlinien stuft die vollständige Resektion von Metastasen als probate Therapieoption mit einem prognostischen Vorteil ein [12].

Wir präsentieren die Ergebnisse der chirurgischen Therapie unserer Patienten mit hepatopankreatischen Metastasen oligometastasierter Nierenzellkarzinome in einer retrospektiven Erfassung und diskutieren die Daten mit den Ergebnissen der Literatur.

## Material und Methoden

Die Daten der Patienten stammen aus dem Tumorregister der Klinik für Allgemein‑, Viszeral- und Gefäßchirurgie des Universitätsklinikums Jena (Ethikvotum 5073-02/17 Ethikkommission der Universität Jena). Die hypervaskularisierten, malignomsuspekten Raumforderungen wurden schnittbildgebend mit Computertomographie oder Magnetresonanztomographie diagnostiziert. Die Indikation zur chirurgischen Therapie erfolgte in den letzten Jahren nach interdisziplinärer Befunddiskussion im pankreatohepatobiliären Tumorboard. Alle Resektionen erfolgten parenchymsparend nach Ausdehnung des Befundes über einen offenen (konventionellen) Zugang. Tumorsuspekte lokale Lymphknoten wurden exstirpiert. Leberteilresektionen führen wir ohne Pringle-Maneuver mit dem CUSA („cavitron ultrasonic aspirator“; CUSA®, Valleylab, Boulder, Colorado, USA) durch.

Zur statistischen Analyse kam SPSS Software Version 19 zur Anwendung. Unterschiede in der Verteilung von Variablen wurden mit dem Mann-Whitney-U-Test oder mit dem χ^2^-Test überprüft. Überlebensraten wurden nach Kaplan-Meier berechnet. Die Signifikanztestung erfolgte mittels Log-Rank-Test.

## Ergebnisse

### Gesamtpatientenkollektiv

Wir haben 18 Patienten mit Pankreas-, 32 Patienten mit Lebermetastasen sowie einen weiteren Patienten mit Metastasen in Leber und Pankreas im Abstand von 2 Jahren operiert. Alle Resektionen im Zeitraum von 1995 bis 2018 erfolgten in kurativer Intention.

Die applizierte Systemtherapie unterlag einem Wandel. Die Tumormanifestationen vor, zum Zeitpunkt und nach der Leber- oder Pankreasresektion aller 52 Fälle sind in Tab. 1 aufgeführt.

**Table 1:** 

Leber		Pankreas	
Lokalisation	Anzahl	Lokalisation	Anzahl
**Tumormanifestationen vor Resektion**			
Lunge	5	Lunge	5
Nebenniere	1	Nebenniere	1
Lokalrezidiv	1	Lokalrezidiv	1
Bauchwand	1	Schilddrüse	1
Dickdarm	1	Hoden	1
Pankreas	1		
**Tumormanifestationen zur Resektion**			
Lunge	7	Nebenniere	2
Nebenniere	1	Peripankreatisch	1
Omentum	3	Peritoneum	1
Lokalrezidiv	3		
Nicht regionäre Lymphknoten	2		
**Tumormanifestationen nach Resektion**			
Leber	11	Leber	2
Lunge	5	Lunge	4
Hirn	1	Skelett	2
Peritoneum	1	Restpankreas	2
		Weichteile	1

Die 5‑Jahres-Überlebensrate aller Patienten nach Leberresektion war statistisch signifikant geringer (38 ± 9 %, mediane Überlebenszeit 34 Monate) als nach Pankreasresektion (69 ± 11 %, mediane Überlebenszeit 69 Monaten; *p* = 0,017). 21 Patienten haben bislang die Metastasenentfernung länger als 5 Jahre überlebt, 4 Patienten länger als 10 Jahre. Die mediane Nachbeobachtungszeit der verstorbenen Patienten beträgt 34 (0–244 Monate), die der noch lebenden 69 (12–174) Monate. Am Ende der Beobachtung waren 37 der 51 Patienten verstorben.

**Figure 1:**
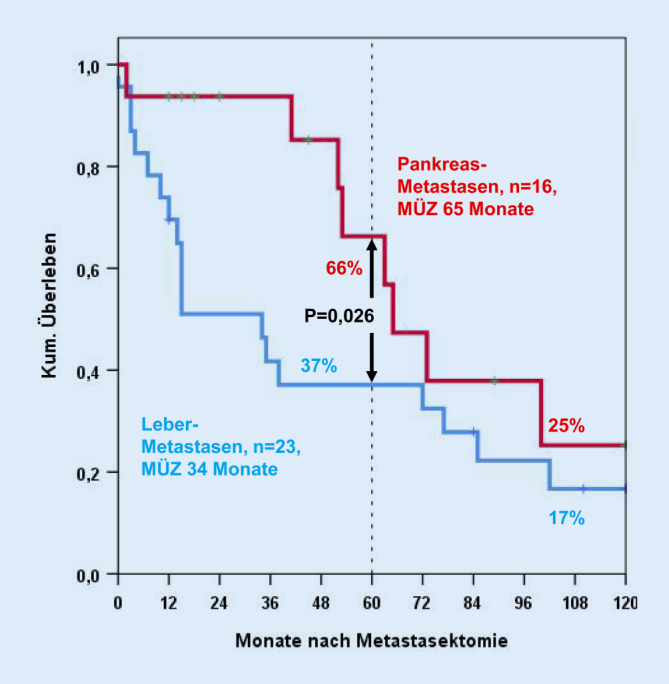

Die 23 Patienten mit einer R0-Leberresektion hatten ein statistisch signifikant schlechteres Langzeitüberleben als die 16 Patienten mit R0-Pankreasresektion (*p* = 0,026). Die 5‑Jahres-Überlebensrate nach Leberresektion war 37 ± 10 % (mediane Überlebenszeit 34 Monate), nach Pankreasresektion 68 ± 13 % (mediane Überlebenszeit 65 Monate; Abb. 1).

### Leber

Von den 33 wegen Lebermetastasen therapierten Patienten erhielten 31 (94 %) Patienten eine Resektion sowie 2 eine Kombination von Resektion und Ablation. Es erfolgte bei 24 (73 %) Patienten eine Major- bzw. bei 9 Patienten (27 %) eine Minorresektion. In der pathologischen Analyse wurde eine R0-, R1- bzw. R2-Resektion bei 23 (70 %), 3 (9 %) bzw. 7 (21 %) Patienten beschrieben.

**Figure 2:**
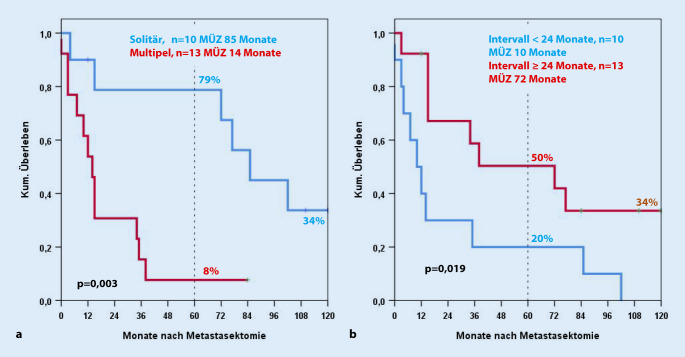

**Figure 3:**
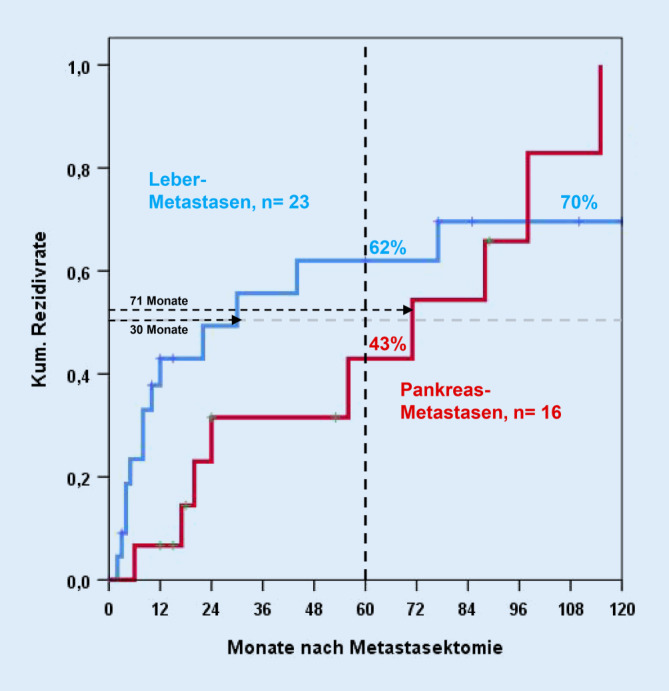

Die Morbidiät (Clavien ≥III) sowie Mortalität der Leberresektionen unserer Studie betrugen 18 % sowie 6 %. 2 Patienten sind innerhalb der ersten 30 Tage postoperativ verstorben. Fünf der leberresezierten Patienten wurden adjuvant mit Sunitinib therapiert. Im metastasierten Stadium mehrere Jahre nach Leberresektion wurden weitere 5 Patienten mit Sunitinib sowie jeweils 2 Patienten chemo- bzw. immuntherapiert. Vor den 33 Lebereingriffen wurden bei 10 Patienten (30 %) andere Rezidive entfernt. Acht der 23 Patienten mit R0-Leberresektion wurden mehrfach an der Leber operiert. Eine Übersicht über die R0-resezierten Patienten zeigt Tab. 2. Bei R0-Leberresektionen erwiesen sich in der univariaten Analyse ein Intervall von <24 Monaten zwischen Nephrektomie und Leberresektion sowie multiple Metastasen als negative Prognosefaktoren (Abb. 2). Extrahepatische Tumorabsiedelungen zum Zeitpunkt des Eingriffs, der Prognose-Score nach Adam et al. [1] sowie vorherige Metastasen hatten dagegen keinen statistisch signifikanten Einfluss auf das Überleben. Die kumulative Rezidivrate nach 5 Jahren betrug bei der Leber 62 % (Abb. 3).

**Table 2:** 

Merkmal		Patientenanzahl	Prozent
*Gesamt*		23	100
*Alter*	Median (Range)	63 (45–78) Jahre	–
*Geschlecht*	Männlich	15	65
	Weiblich	8	35
*Leber MTS*	Synchron	4	17
	Metachron	19	83
*Anzahl MTS*	Solitär	10	43
	Multipel	13	57
*MTS vorher*		7	30
*Extrahepatischer Tumor*		8	35
*Tumorrezidiv*		13	57
*Zweittumoren*	Synchron	0	0
	Vorher	1	4
	Im Verlauf	5	22
*Leberresektion*	<3 Segmente	7	30
	≥3 Segmente	16	70
*Nachbeobachtung*	Median (Range)	15 (0–244) Monate	–
*Komplikationen Clavien ≥III (30* *Tage)*		3	13
*Todesfälle (30* *Tage)*		1	4

*MTS* Metastasen

### Pankreas

Von den 19 Patienten mit Pankreasmetastasen wurde bei 3 Patienten eine Pankreaskopfresektion, bei 4 Patienten eine Pankreatektomie sowie bei den übrigen 12 Patienten eine Pankreaslinksresektion durchgeführt. Drei pankreasresezierte Patienten wurden adjuvant mit Sunitinib therapiert. In der pathologischen Untersuchung wurde eine R0- bzw. R1-Resektion bei 16 (84 %) bzw. 3 (16 %) Patienten beschrieben. Kein Patient war postoperativ verstorben, die Morbidität (Clavien ≥III) betrug 31 %. Eine Übersicht über die R0-resezierten Patienten zeigt Tab. 3. Die kumulative Rezidivrate nach 5 Jahren betrug beim Pankreas 43 % (Abb. 3).

**Table 3:** 

Merkmal		Patientenanzahl	Prozent
*Gesamt*		16	100
*Alter*	Median (Range)	71 (36–79) Jahre	–
*Geschlecht*	Männlich	9	56
	Weiblich	7	44
*Leber MTS*	Synchron	0	0
	Metachron	16	100
*Anzahl MTS*	Solitär	13	81
	Multipel	3	19
*MTS vorher*		8	50
*Extrapankreatischer Tumor*		3	19
*Tumorrezidiv*		9	56
*Zweittumoren*	Synchron	2	13
	Vorher	3	19
	Im Verlauf	1	6
*Nachbeobachtung (Monate)*	Median (Range)	60 (2–156)	–
*Pankreatektomie*		4	25
*Pankreaskopfresektion*		3	19
*Pankreaslinksresektion*		9	56
*Komplikationen Clavien ≥III (30* *Tage)*		5	31
*Todesfälle (30* *Tage)*		0	0

*MTS* Metastase

## Diskussion

Der Stellenwert der chirurgischen Therapie oligometastasierter Tumorerkrankungen wird aktuell diskutiert. Die vorliegenden Studien haben einen unterschiedlichen Case-Mix insbesondere auch im Hinblick auf potenzielle Prognosefaktoren (Tab. 4). So unterlag die Systemtherapie im Untersuchungszeitraum einem zeitlichen Wandel. Dies erschwert die Wertung der Therapieergebnisse.

**Table 4:** 

	Jahr	Pat	MTS vorher (%)	MTS synchron (%)	Tumorfreies Intervall	EHT/EPT (%)	Solitär (%)	R0-reseziert (%)	Mortalität (%)	Morbidität Clavien ≥III (%)	MÜZ (Mo)	5‑JÜR (%)
*Leber*												
Aloia et al. [3]	2006	19	37	26	53 Mo	37	42	89	5,3	32	36	26
Thelen et al. [23]	2007	31	19	19	kA	10	kA	77	3,2	12,9	48	39
Staehler et al. [20]	2010	68	kA	28	kA	0	38	kA	0	20	142	62
Ruys et al.^a^ [16]	2011	33^c^	kA	35	50 Mo (7–360)	33	66	76	0	18	33	43
Hatzaras et al.^a^ [9]	2012	43^f^	0	21	17 Mo (2–189)	33	26	95	2	23 %	>60	62
Langan et al.^a^ [11]	2012	18^d^	kA	28	54 Mo (12–144)	22	78	100	0	30	24	34
Hau et al. [10]	2016	35^g^	kA	17	15 Mo (3–39)	20	kA	86	0	20 %	kA	51
Beetz et al. [5]	2020	40	kA	8	44 Mo (3–278)	35	kA	92	2	23	38	≈38
Eigene Daten	2020	33 ^e^	30 %	15	31 Mo (9–183)	48	36	70	6	18	34	41
*Pankreas*												
Zerbi et al. [30]	2008	23	23	0	8 Jahre	48	65	kA	0	48	kA	88
Tanis et al.^b^ [22]	2009	321	10	8	10 Jahre	9	65	kA	3	kA	69	67
Yazbek u. Gayet [27]	2012	11	18	9	≈11 Jahre	0	100	90	1	55	78	kA
Schwarz et al.^a^ [18]	2014	62	13	3	10 Jahre	10	63	kA	6	kA	kA	63
Tosoian et al. [24]	2014	42	kA	10	11 Jahre	19	kA	88	5	19	kA	50
Fikatas et al. [7]	2016	19	kA	0	≈10 Jahre	22	kA	kA	0	21	kA	71
Madkhali et al. [13]	2018	17	kA	35	kA	35	kA	100	0	n. s.	kA	47
Eigene Daten	2020	19	47	5	13 Jahre	21	74	84	0	31	69	73

*MTS* Metastasen, *Mo* Monate, *EHT* extrahepatischer Tumor bei Leberresektion, *EPT* extrapankreatischer Tumor bei Pankreasresektion, *MÜZ* mediane Überlebenszeit nach Metastasektomie, *kA* keine Angabe, *JÜR* Jahresüberlebensrate

^a^Multizentrische Studie

^b^Review

^c^4 Patienten Radiofrequenzablation

^d^9 Patienten Radiofrequenzablation

^e^2 Patienten Resektion + Radiofrequenzablation, 1 Patient selektive interne Radiotherapie

^f^7 Patienten Radiofrequenzablation

^g^2 Patienten Radiofrequenzablation

### Leber

Mit Senkung der perioperativen Morbidität und Mortalität (Tab. 4) wird über die Indikationsstellung der Metastasenchirurgie nichtkolorektaler Tumoren an der Leber und die sinnvolle Auswahl der Patienten nach Art des Primarius und der Tumorlast diskutiert [1]. Die Analyse ist infolge der langen Krankheitsverläufe sowie der Anwendung verschiedener Systemtherapien komplex. Das tumorfreie Intervall bis zum Auftreten von Lebermetastasen ist mit etwa 5 Jahren sehr viel kürzer als das tumorfreie Intervall bis zum Auftreten von Pankreasmetastasen mit etwa 10 Jahren (Tab. 4). In der untersuchten Literatur variierte der Anteil von Patienten mit Metastasen vor der Leberresektion zwischen 0 und 37 %, der Anteil mit synchronen Metastasen zum Zeitpunkt der Leberresektion zwischen 8 und 35 %, der Anteil solitärer Tumoren zwischen 26 und 78 % sowie der Anteil der R0-Resektionen zwischen 76 und 100 % (Tab. 4). Der Vergleich der Ergebnisse ist schwierig, da der Einfluss der dargestellten Parameter auf die Prognose unklar ist. In der größten Untersuchung von Adam et al. wird für 85 Patienten mit Lebermetastasen eines Nierenkarzinoms eine 5‑Jahres-Überlebensrate von 38 % angegeben, jedoch fehlen die Angaben zu prognostischen Parametern [1].

Das Langzeitüberleben unserer R0-leberteilresezierten Patienten wurde in der univariaten Analyse statistisch signifikant von der Anzahl der Lebermetastasen sowie dem Intervall zwischen Nephrektomie und Leberresektion beeinflusst.

In der Literatur beeinflussten die R‑Klassifikation des Lebereingriffs [10, 16, 23], das Grading [20], das Intervall zwischen Nephrektomie und Diagnose der Metastase [3, 5, 10, 11, 16, 20, 23], andere Tumormanifestationen vor der Lebermetastase [9], der Nachweis eines extrahepatischen Tumors zum Zeitpunkt der Leberresektion [5, 9], die Metastasengröße [10], der Performance-Status des Patienten sowie eine Systemtherapie [10] die Prognose nach Leberteilresektion.

Das zeitliche Auftreten der Metastasen – synchron, Frühmetastasen sowie Spätmetastasen – wird unterschiedlich definiert. Nur Hatzaras et al. [9] sahen keinen statistisch signifikanten Einfluss des Intervalls zwischen Resektion des Primarius und der Metastasektomie auf das Überleben (Tab. 4).

In den Arbeiten von Hatzaras et al., Hau et al., Beetz et al. sowie Staehler et al. wurden die wegen Lebermetastasen von Nierenzellkarzinomen operierten Patienten zwischen 37 und 80 % mit Immun- bzw. Chemotherapie behandelt [5, 9, 10, 20]. Beetz et al. und Hau et al. sahen ein statistisch signifikant besseres Überleben bei den mit Tyrosinkinaseinhibitor (Sunitinib) therapierten Patienten (*p* = 0,038; [5, 10]). Hau et al. sahen außerdem bei einem ECOG(Eastern Cooperative Oncology Group)-Status >1 sowie synchronen Lebermetastasen einen negativen Einfluss auf das Überleben [10]. Die mediane Überlebenszeit variierte in der Literatur zwischen 24 und 142 Monaten (Tab. 4).

Auffallend sind die guten Langzeitergebnisse von Staehler et al. [20]. In dieser Studie wurden jedoch Patienten mit extrahepatischem Tumor ausgeschlossen und die Mehrzahl der Patienten adjuvant therapiert. Die Autoren sahen einen Überlebensvorteil bei den Patienten mit Metastasektomie (*p* = 0,087; [20]). Auch Pinotti et al. bestätigten im Rahmen eines systematischen Reviews von 378 Patienten mit Lebermetastasen von Nierenzellkarzinomen den prognostischen Vorteil einer chirurgischen Therapie auf das Überleben. Die Einbindung der Patienten in multimodale Therapiekonzepte ermöglicht die Identifikation der Patienten, welche im metastasierten Tumorstadium einen realen Benefit von der Chirurgie haben, wobei die Reihenfolge von Target-Therapie und Chirurgie noch nicht abschließend geklärt ist [15].

### Pankreas

Metastasenchirurgie des Pankreas ist deutlich seltener. Pankreasresektionen wegen Metastasen erfolgen bei ca. 2–5 % aller Pankreasresektionen [4]. Der Anteil von Metastasen von Nierenzellkarzinomen beträgt 50–60 % [19]. Eine niedrige perioperative Mortalität und Morbidität sind dafür die unabdingbare Voraussetzung (Tab. 4). In der Literatur beeinflussten eine Gefäßinvasion [24], eine Tumormanifestation vor der Pankreasmetastase [18], der Nachweis eines extrapankreatischen Tumors [22], das Intervall zwischen Nephrektomie und Pankreasresektion [22] sowie eine symptomatische Erkrankung [24] die Prognose nach Pankreasresektion.

Vorangegangene Metastasenchirurgie war kein negativer Prognosefaktor [22, 30]. Neben dem perioperativen Risiko muss der Langzeiterfolg im Vergleich zur Systemtherapie sowie die Option anderer wirksamer Therapien in Kombination mit einem chirurgischen Eingriff berücksichtigt werden [21]. In der Studie von Grassi et al. überlebten die Patienten mit operativer Therapie länger als die systemisch therapierten Patienten (106 bzw. 59 Monate; [8]). In der retrospektiven multizentrischen Studie von Santoni et al. fand sich kein statistisch signifikanter Unterschied im medianen Überleben (Operation vs. Tyrosinkinaseinhibitor 103 vs. 86 Monate). Jedoch muss man die chemotherapiebedingten Nebenwirkungen dem perioperativen Risiko gegenüberstellen [17]. Lediglich in der Arbeit von Schwarz et al. [18] wurden 5 % der wegen Pankreasmetastasen operierten Patienten adjuvant systemisch therapiert.

Randomisierte prospektive Studien zum Stellenwert der Metastasenresektion resektabler Pankreastumoren sind bei dieser seltenen Tumorentität schwer vorstellbar.

### Stellenwert der kompletten Metastasektomie

Es existieren bisher keine randomisierten Studien zum Stellenwert der kompletten Metastasektomie beim metastasierten Nierenzellkarzinom. In dem systematischen Review von 16 Studien von Dabestani et al. [6] sowie dem systematischen Review und der Metaanalyse (2267 Patienten) von Zaid et al. [29] profitierten die Patienten von einer kompletten Metastasektomie mit einem verbesserten Überleben sowie einer Symptomkontrolle. Allerdings betrachten die Autoren überwiegend Lungen‑, Knochen- und Hirnmetastasen, Patienten mit hepatopankreatische Metastasen sind sehr selten Auch wenn die Auswahl keine Aussage für einzelne Tumorlokalisationen zulässt, so zeigt sich ein allgemeiner Trend für die chirurgische Therapie. Für metachrone hepatopankreatische Metastasen bei Patienten in gutem Allgemeinzustand wird unter Abwägung der perioperativen Risiken die operative Therapie, ggf. in Kombination mit lokal ablativen Therapien, empfohlen. Dagegen sollte bei Patienten mit eingeschränktem Allgemeinzustand sowie kurzem Intervall zwischen Nephrektomie und Manifestation der Metastase eher eine Systemtherapie erwogen werden [6]. In der Ära der Target-Therapie beobachteten Yu et al. ein medianes Überleben von 52, 16 bzw. 22 Monaten bei kompletter, inkompletter Metastasektomie bzw. Target-Therapie (mTOR-Inhibitor, Multikinaseinhibitor). Die Patienten mit einer kompletten Metastasektomie hatten ein statistisch signifikant besseres Überleben als die Nichtoperierten. Das Überleben bei inkompletter Metastasektomie bzw. Target-Therapie war nicht statistisch signifikant unterschiedlich [28].

## Schlussbetrachtung

Patienten mit Pankreasmetastasen hatten ein besseres Überleben als Patienten mit Lebermetastasen eines Nierenzellkarzinoms. In unserer Studie konnte durch einen chirurgischen Eingriff, ggf. in Kombination mit einem lokal ablativen Verfahren, der Krankheitsverlauf sowie das Überleben der Patienten mit hepatopankreatischen Metastasen von Nierenzellkarzinomen positiv beeinflusst werden.

## Fazit für die Praxis

- Aus unserer Studie resultieren Hinweise, dass die Prognose von Patienten mit hepatopankreatischen Metastasen oligometastasierter Nierenzellkarzinome durch Metastasektomie verbessert werden kann.
- Inwieweit neoadjuvante/adjuvante Systemtherapien die Überlebens- und Rezidivraten verbessern können, bleibt abzuwarten.
- Es wären prospektive Studien zur Evaluierung des Stellenwertes der neo- sowie adjuvanten Systemtherapie wünschenswert.
